# Inhibitory effects of Tomivosertib in acute myeloid leukemia

**DOI:** 10.18632/oncotarget.27952

**Published:** 2021-05-11

**Authors:** Milagros Suarez, Gavin T. Blyth, Alain A. Mina, Ewa M. Kosciuczuk, Blazej Dolniak, Shira Dinner, Jessica K. Altman, Elizabeth A. Eklund, Diana Saleiro, Elspeth M. Beauchamp, Leonidas C. Platanias

**Affiliations:** ^1^Robert H. Lurie Comprehensive Cancer Center of Northwestern University, Chicago, IL, USA; ^2^Division of Hematology/Oncology, Feinberg School of Medicine, Northwestern University, Chicago, IL, USA; ^3^Division of Hematology-Oncology, Department of Medicine, Jesse Brown Veterans Affairs Medical Center, Chicago, IL, USA

**Keywords:** MNK, Tomivosertib, acute myeloid leukemia, eIF4E

## Abstract

The MAPK-interacting kinases 1 and 2 (MNK1/2) have generated increasing interest as therapeutic targets for acute myeloid leukemia (AML). We evaluated the therapeutic potential of the highly-selective MNK1/2 inhibitor Tomivosertib on AML cells. Tomivosertib was highly effective at blocking eIF4E phosphorylation on serine 209 in AML cells. Such inhibitory effects correlated with dose-dependent suppression of cellular viability and leukemic progenitor colony formation. Moreover, combination of Tomivosertib and Venetoclax resulted in synergistic anti-leukemic responses in AML cell lines. Mass spectrometry studies identified novel putative MNK1/2 interactors, while in parallel studies we demonstrated that MNK2 - RAPTOR - mTOR complexes are not disrupted by Tomivosertib. Overall, these findings demonstrate that Tomivosertib exhibits potent anti-leukemic properties on AML cells and support the development of clinical translational efforts involving the use of this drug, alone or in combination with other therapies for the treatment of AML.

## INTRODUCTION

Acute myeloid leukemia (AML) is the second most common form of leukemia in adults, and has a very poor overall survival rate [1, 2]. For many years, chemotherapy based on the administration of anthracycline/cytarabine combinations has been the mainstay of AML treatment [3, 4]. More recently, advancements in next-generation sequencing technologies and understanding of genomic alterations involved in leukemogenesis have allowed the development of several novel targeted therapy approaches that complement the action of the currently available chemotherapeutic treatments. Among them are targeted drugs for specific mutations found in AML, such as the FLT3 inhibitors, IDH1/IDH2 inhibitors, pro-apoptotic agents, Hedgehog pathway inhibitors and others [5–7]. Even though these therapies have increased at variable degrees the response rates and survival benefit of subgroups of AML patients [8–11]; the development of resistance towards these novel drugs and subsequent relapse remains one of the major challenges for the treatment of this disease [12]. Therefore, there continues to be a need for new therapeutic modalities, including approaches targeting negative-feedback signaling pathways that may be activated in response to antileukemic treatments, leading to resistance.

The mitogen-activated protein kinase (MAPK) signaling pathway is a major regulatory cascade, activated in 70–80% of AML patients [13, 14]. MAPK-interacting kinases 1 and 2 (MNK1/2) are downstream effectors of this pathway, which control the activation of the eukaryotic translation initiation factor 4E (eIF4E) [15–17]. eIF4E has been demonstrated to be overexpressed in AML [18, 19] and has been linked to malignant cell transformation and proliferation [19–23]. The pro-neoplastic activity of eIF4E is associated with its phosphorylation/activation by MNK1/2 on serine 209 [24–26] and correlates with enhanced mRNA translation, as well as nuclear export of mRNAs involved in tumorigenesis and cell cycle control [26–31]. Several studies have shown that pharmacological targeting of MNK1/2 results in inhibitory activity against AML cells in pre-clinical models [32–37]. However, the extent of these effects has been frequently demonstrated using MNK1/2 inhibitors that lack high selectivity against MNK1/2 [32–37]. As a result, the full therapeutic potential of MNK1/2 inhibition for the treatment of AML has not been fully assessed. It should be noted that in addition to targeting eIF4E, MNK1/2 has been reported to phosphorylate other mRNA binding proteins such as the heterogeneous nuclear ribonucleoprotein A1 (hnRNPA1) [38] and the polypyrimidine tract-binding protein-associated splicing factor (PSF) [39]. However, only a few additional substrates of MNK1/2 have been identified beyond proteins involved in mRNA translation regulation, including cytosolic phospholipase A2 (cPLA2) [40] involved in arachidonate release, and Sprouty2 (Spry2) [41], which modulates signaling downstream of receptor tyrosine kinases. Therefore, identification of new components of the MNK1/2 pathway that could serve as potential targets may provide valuable information with therapeutic potential.

Tomivosertib, also known as eFT-508, is a potent, highly selective and orally bioavailable MNK1 and MNK2 inhibitor that is currently under investigation in Phase 1/2 clinical trials for the treatment of patients with advanced solid tumors and lymphomas [42]. This drug inhibits the activity of MNK1 and MNK2 with half-maximal inhibitory concentration (IC_50_) values of 2.4 nM and 1 nM; and has demonstrated potent anti-neoplastic effects against several cell lines and tumor models including diffuse large B-cell lymphoma (DLBCL), breast cancer, and lung cancer [42]. However, its potential activity against AML has not been explored. In this study, we investigated the efficacy of Tomivosertib in pre-clinical models of AML. We demonstrate that Tomivosertib suppresses eIF4E phosphorylation in AML cells and decreases leukemic cell survival and proliferation. We also provide evidence for synergy of Tomivosertib with Venetoclax, *in vitro*.

## RESULTS

### Tomivosertib inhibits eIF4E phosphorylation and decreases leukemic cell survival and proliferation

**Figure 1:**
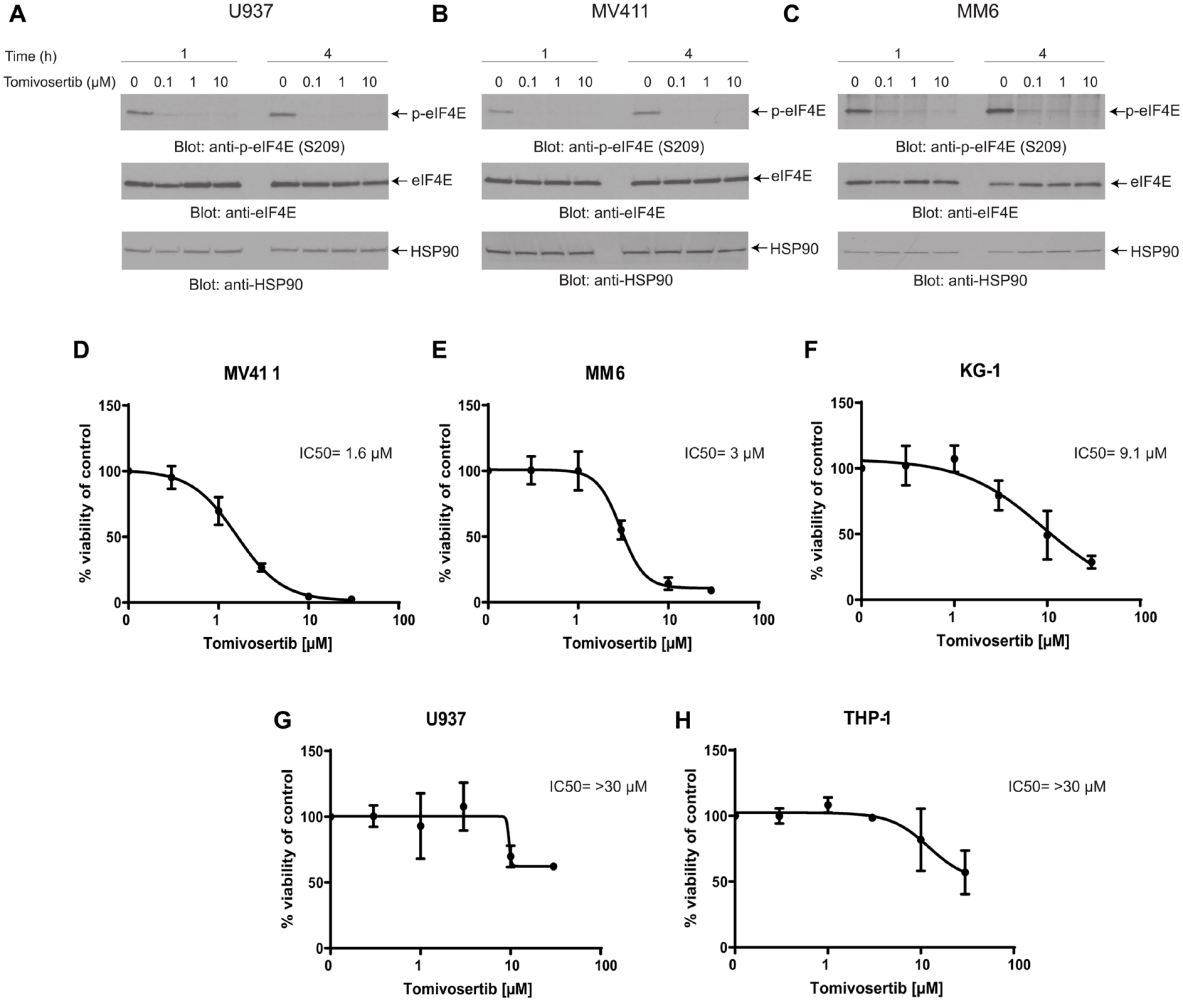
Tomivosertib blocks phosphorylation of eIF4E at Ser209 and inhibits cell viability in AML cells. (**A**) U937, (**B**) MV411, (**C**) MM6 cells were incubated with Tomivosertib for 1 hour and 4 hours at final concentrations of 0.1, 1 or 10 μM. Equal amounts of total cell lysates were resolved by SDS-PAGE and immunoblotted with the indicated antibodies. Membranes were probed with the p-eIF4E S209 antibody and then stripped and reprobed for total eIF4E. (**D**) MV411, (**E**) MM6, (**F**) KG-1, (**G**) U937 and (**H**) THP-1 cells were treated with increasing concentrations of Tomivosertib for 4 days. Viability was assessed using a WST-1 assay. Data are expressed as a percentage of control (DMSO-treated) cells. The means ± SE of three independent experiments, each done in triplicate, and IC50 values are shown for each cell line.

**Figure 2:**
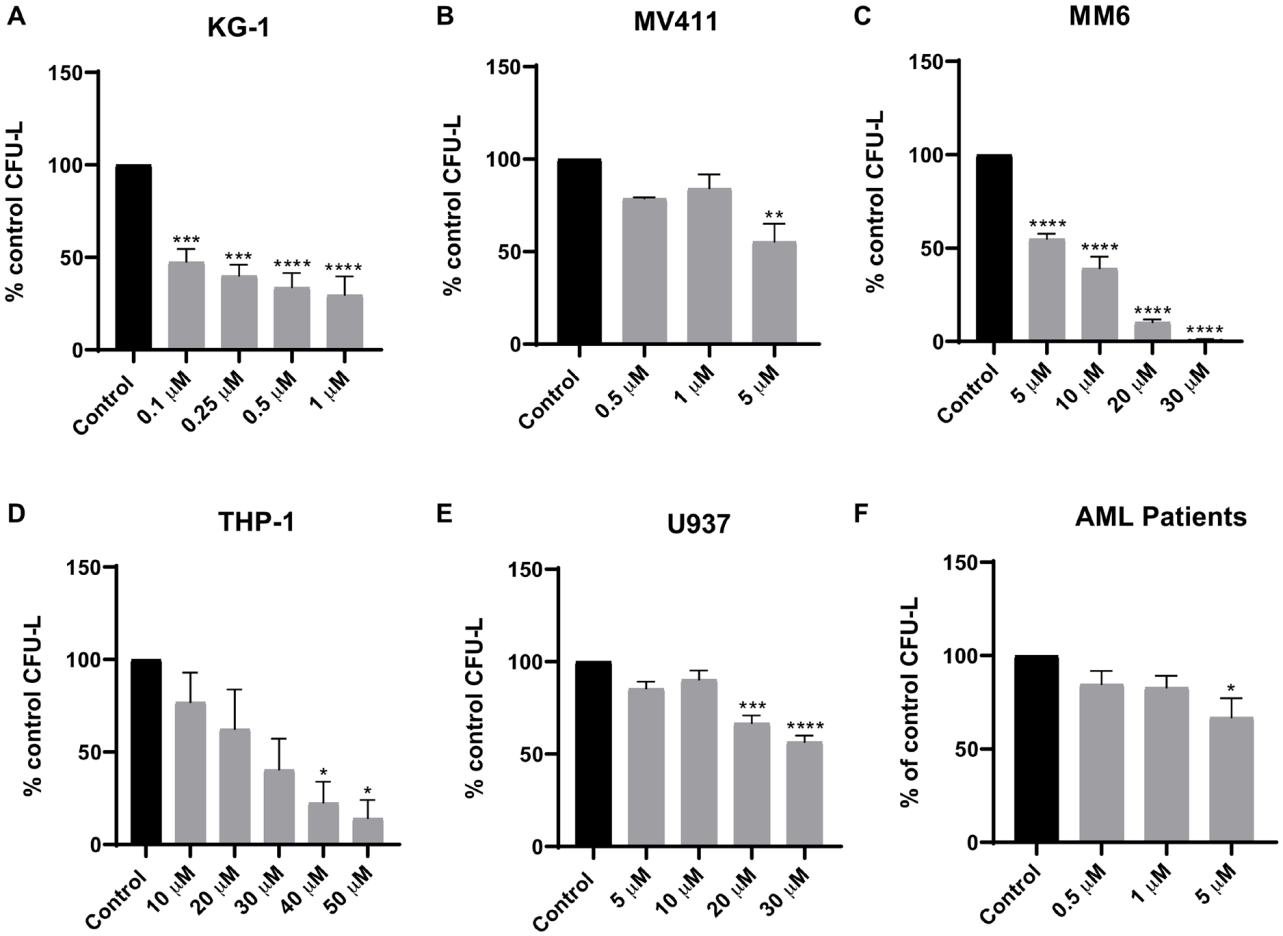
Tomivosertib suppresses growth of AML leukemic progenitors. The effects of Tomivosertib on leukemic-progenitors of (**A**) KG-1, (**B**) MV411, (**C**) MM6, (**D**) THP-1 and (**E**) U937 cells were assessed in clonogenic assays in methylcellulose. Data are expressed as percentage of CFU-L of control (DMSO-treated) cells and represent means ± SE of three independent experiments for U937, MV411, MM6 and THP-1 and four independent experiments for KG-1. (**F**) The inhibitory effects of Tomivosertib on primary leukemic progenitors from AML patients were assessed in clonogenic assays in methylcellulose. Data are expressed as percentage of CFU-L of control (DMSO-treated) and represent means ± SE from three independent experiments, using cells from three different patients with AML. One-way ANOVA analysis followed by Tukey’s test was used to evaluate statistically significant differences: ^*^ *p* < 0.05, ^**^ *p* < 0.01, ^***^ *p* < 0.001, ^****^ *p* < 0.0001.

Initially, we determined whether Tomivosertib blocks phosphorylation of the MNK effector eIF4E in AML cells. U937, MV411 and MM6 cells were treated with increasing doses of Tomivosertib for a period of 1 and 4 hours and eIF4E phosphorylation was assessed. Treatment with Tomivosertib abrogated the phosphorylation of eIF4E on serine 209 at a concentration of 0.1 μM, supporting potent inhibition of MNK1/2 activity in AML cells (Figure 1A–1C). To determine the potential antileukemic effects of Tomivosertib, we employed cellular viability assays as well as clonogenic assays in methylcellulose, using a panel of leukemia cell lines. Tomivosertib treatment resulted in dose-dependent inhibition of cellular viability in the AML lines tested. The compound showed the greatest suppressive activity against MV411, MM6 and KG-1 cells (Figure 1D–1F). Conversely, higher concentrations of the drug were required to suppress the viability of the myelomonocytic THP-1 and U937 cell lines (Figure 1G and 1H). A similar pattern was observed when the effects of Tomivosertib on leukemic progenitor cells were assessed. Treatment with Tomivosertib significantly inhibited KG-1-, MV411-, and MM6-derived leukemic progenitor (CFU-L) colony formation, while the effects were less pronounced on THP1- and U937-derived colony formation (Figure 2A–2E). Inhibitory effects were also seen on primary leukemic precursors from AML patients (Figure 2F). Taken together, these studies demonstrate that Tomivosertib inhibits eIF4E phosphorylation in AML cells, resulting in decreased leukemic cell survival and proliferation.

### Tomivosertib synergistically enhances the anti-leukemic effects of Venetoclax against AML cells *in vitro*

**Figure 3:**
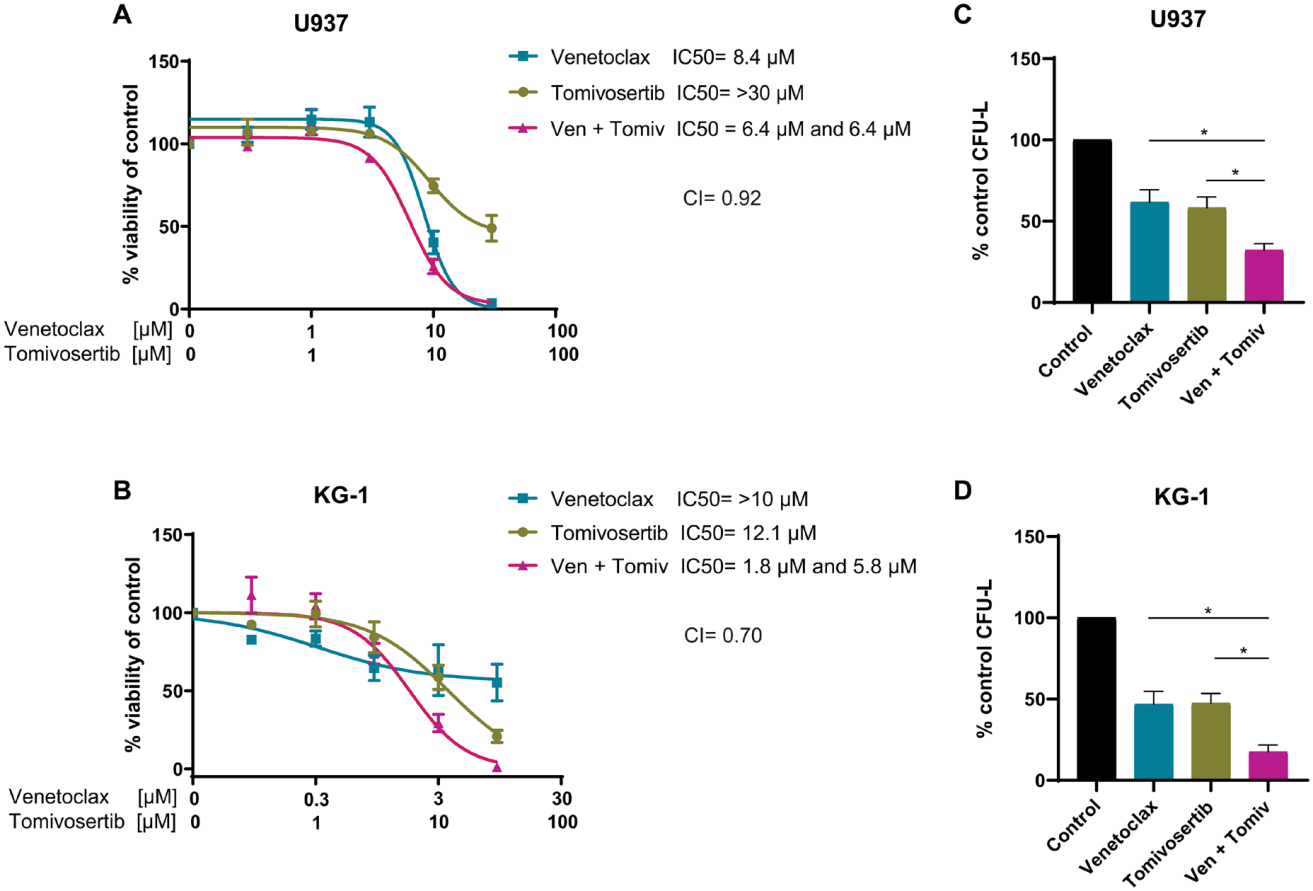
Tomivosertib synergizes with Venetoclax and enhances its anti-leukemic effects *in vitro*. (**A**) U937 and (**B**) KG-1 cells were treated with increasing concentrations of Tomivosertib and/or Venetoclax for 4 days. Viability was assessed using WST-1 assay. Data are expressed as percentage of control (DMSO-treated) cells. Shown are the means ± SE of three independent experiments. (**C**) U937 a were plated in methylcellulose with 30 μM Tomivosertib and 30 μM Venetoclax alone or in combination, as indicated. Data are expressed as percentage of colony formation of control (vehicle-treated) cells and represent means ± SE of four independent experiments. One-way ANOVA analysis followed by Tukey’s test was used to evaluate statistically significant differences: ^*^ *p* < 0.05. (**D**) KG-1 cells were plated in methylcellulose with 100 nM Tomivosertib and 100 nM Venetoclax alone or in combination, as indicated. Data are expressed as percentage of colony formation of control (vehicle-treated) cells and represent means ± SE of three independent experiments. One-way ANOVA analysis followed by Tukey’s test was used to evaluate statistically significant differences: ^*^ *p* < 0.05.

In consequent studies, we investigated the antileukemic activity of a combined treatment of Tomivosertib with Venetoclax in AML cell lines. Venetoclax is a novel BCL-2 inhibitor that was recently approved by the FDA for the treatment of elderly patients with AML due to its significant clinical activity in combination with cytarabine or hypomethylating agents [43, 44]. However, the development of resistance to Venetoclax can occur through upregulation of other pro-survival proteins such as Myeloid cell leukemia 1 (MCL-1) [45, 46]. MNK1/2 was previously shown to be required for mRNA translation of MCL-1 [24, 33], and therefore, we hypothesized that concomitant treatment of cells with Venetoclax and a MNK1/2 inhibitor may help to overcome the resistance to this drug. We first investigated the effects of combination treatment with Tomivosertib and Venetoclax on cellular viability of different AML cell lines. The combination of Tomivosertib with Venetoclax resulted in synergistic inhibitory effects on cellular viability (Figure 3A and 3B). Similarly, the combination of Tomivosertib and Venetoclax resulted in potent inhibition of U937- and KG-1-derived CFU-L growth as compared to each agent alone (Figure 3C and 3D). Altogether, these studies provide evidence that the Tomivosertib/Venetoclax combination induces potent anti-leukemic responses.

### LC-MS/MS analysis identifies putative MNK1/2 targets and interactors

**Figure 4:**
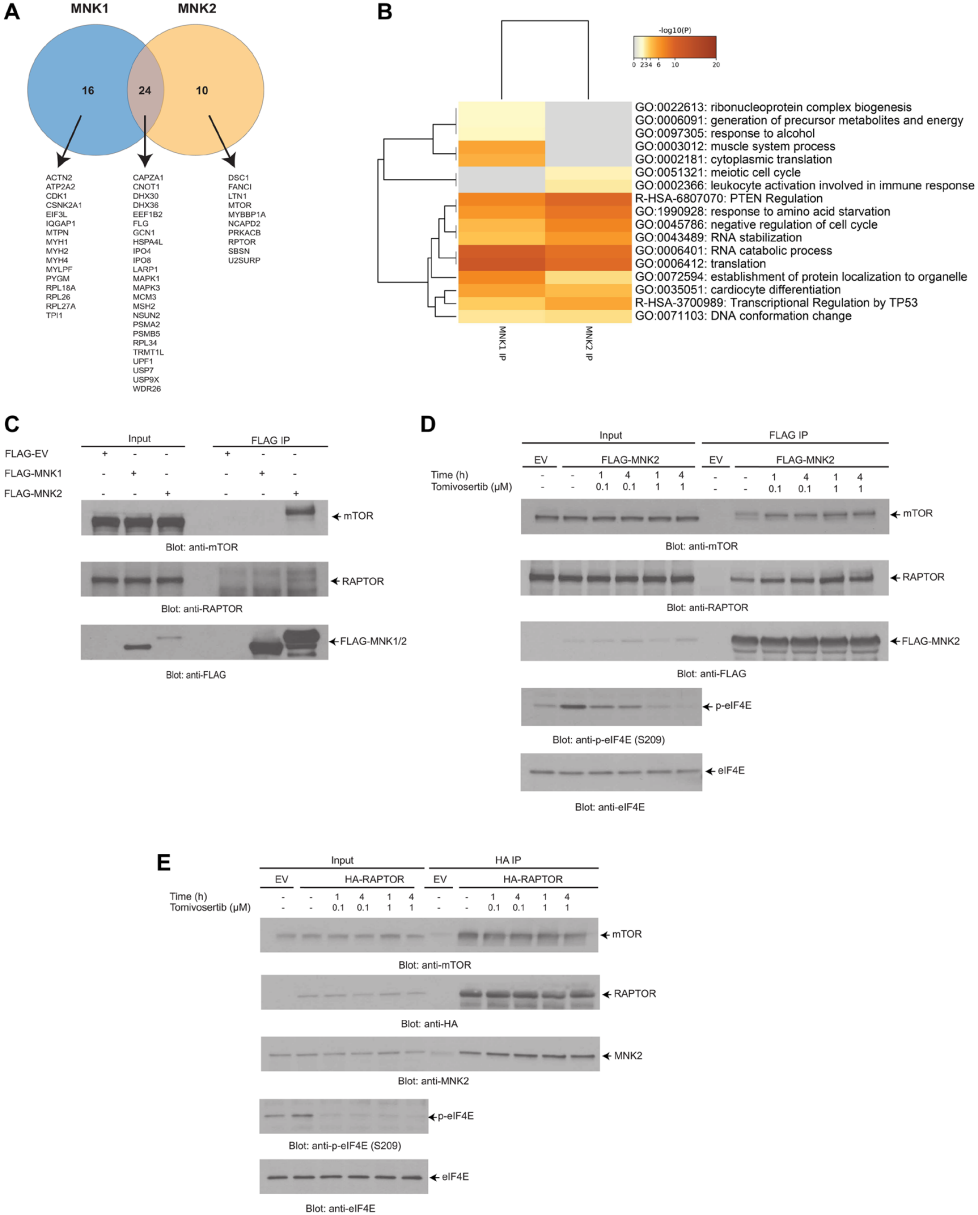
LC-MS/MS analysis identifies putative MNK1/2 targets and interactors. (**A**) FLAG-MNK2 or FLAG-MNK1 was overexpressed in 293T cells. MNK1/2 was immunoprecipitated from 293T cell lysates using anti-FLAG-M2 agarose conjugated beads. An empty vector was used as a negative control. Immunoprecipitated proteins were resolved by SDS-PAGE, and were prepared using standard techniques and then analyzed via LC-MS/MS. A Venn diagram was created depicting the number of proteins that interact with MNK1/2. (**B**) The results from (A) were annotated using Metascape. The heat map shows the most significant pathways and the overlap between the MNK1 IP and MNK2 IP. (**C**) FLAG-MNK2 or FLAG-MNK1 was immunoprecipitated as described in (A). Proteins were resolved by SDS-PAGE and immunoblotted with the indicated antibodies. (**D**) FLAG-MNK2 was overexpressed in 293T cells. Cells were treated with either DMSO (vehicle-control) or Tomivosertib at the indicated doses and time points, lysed and MNK2 was immunoprecipitated with anti-FLAG-M2 agarose conjugated beads. An empty vector (EV) was used as a negative control. Proteins were resolved by SDS-PAGE and immunoblotted with the indicated antibodies. (**E**) HA-RAPTOR was overexpressed in 293T cells. Cells were treated with either DMSO (vehicle-control) or Tomivosertib at the indicated doses and time points, lysed and RAPTOR was immunoprecipitated with anti-HA Sepharose conjugated beads. An empty vector (EV) was used as negative control. Proteins were resolved by SDS-PAGE and immunoblotted with the indicated antibodies. (C–E) Total cell lysates for each experimental condition were run in parallel with the immunoprecipitated proteins (Input).

To identify novel binding partners of MNK1/2, liquid-chromatography-tandem mass spectrometry (LC-MS/MS) analysis was performed on protein-MNK1/2 complexes immunoprecipitated from 293T cells. We found that 16 proteins interacted with MNK1, 10 proteins interacted with MNK2 and 24 proteins interacted with both MNK1 and MNK2 (Figure 4A). Pathway and process enrichment analysis of the putative MNK1/2 interactors identified translation as one of the most significantly represented pathway activity, consistent with the role of MNK1/2 in this process (Figure 4B).

Among the different proteins, RAPTOR and mTOR were identified as interacting partners of MNK2 in the LC-MS/MS studies (Figure 4A). We first confirmed the interaction between MNK2, mTOR and RAPTOR observed in the proteomic analysis. For this purpose, we overexpressed FLAG-MNK2 and FLAG-MNK1 in 293T cells and assessed the ability of MNK1 or MNK2 to interact with RAPTOR and mTOR by co-immunoprecipitation. Only MNK2 interacted with both RAPTOR and mTOR (Figure 4C). We then determined whether this interaction between MNK2 and RAPTOR was altered by treatment with Tomivosertib (Figure 4D and 4E). We also then further confirmed this interaction by overexpressing HA-RAPTOR and assessed the ability of RAPTOR to bind to MNK2 by co-immunoprecipitation (Figure 4E). Together, these findings indicate that MNK2 interacts with mTORC1 but that Tomivosertib does not affect the interaction.

## DISCUSSION

Protein synthesis is commonly dysregulated in solid tumors and hematological malignancies [47, 48]. A key regulatory node in this process is the cap-binding complex that includes eIF4E, which controls translation initiation of many oncogenic mRNAs [26–31]. Both, overexpression of eIF4E or its phosphorylation on serine 209 by MNK1/2 have been implicated in cellular transformation [22, 24–28] and are associated with poor prognosis in a wide range of malignancies, among them AML [18, 19]. Due to the highly important role of eIF4E in tumorigenesis, several approaches to inhibit its function and/or target its expression have been developed, including anti-sense oligonucleotides, as well as drugs that mimic the 7-methylguanosine (m^7^G) cap or prevent the interaction of eIF4E with other proteins in the cap-binding complex [18, 49–55]. An alternative means to target oncogenic eIF4E activity, is by preventing its phosphorylation by MNK1/2 [24, 25, 56]. Given that the MNK1/2-induced eIF4E phosphorylation modulates the translation of mRNAs with tumorigenic potential rather than global protein synthesis [21, 25, 57–60]; and that MNK1/2 is dispensable for normal cell growth and development including normal hematopoiesis [61]; this strategy may constitute a safer and more specific option for cancer treatment due to a lack of toxicity on normal cells. Moreover, inhibition of the MNK1/2-eIF4E signaling axis results in robust anti-tumor responses, both *in vitro* and *in vivo* [21, 32–37].

In the current study, we assessed the anti-leukemic effects of Tomivosertib [42], a highly selective MNK1/2 inhibitor that has recently entered phase 1/2 clinical trials for the treatment of patients with several types of solid tumors and lymphomas. Our studies demonstrate that Tomivosertib induces potent suppressive effects on leukemic cell viability and clonogenicity through inhibititon of eIF4E phosphorylation on serine 209 in different AML cell lines. Similar to what was observed with other less potent or specific MNK inhibitors; Tomivosertib was most effective at suppressing the growth of M5 subtype AML cells that acquire FLT3 activating mutations, such as MV4-11 and MM6 [32–36]. This is consistent with the fact that the M5 subtype of AML is characterized by the expression of high levels of phosphorylated eIF4E, which correlates with poor prognosis in this cancer [18, 19]. Moreover, the MAPK signaling cascade can be activated downstream of the FLT3 receptor [62]. Therefore, the concomitant presence of mutant proteins upstream of MNK1/2 such as FLT3 and elevated eIF4E levels, may constitute the best model for exploring the potential clinical utility of Tomivosertib. In contrast, THP-1, U937 and KG-1 cells, which do not express FLT3 mutations, were less sensitive to the compound. These cell lines, however, all have p53 mutations which results in loss of function of p53. In our LC/MS/MS analysis of proteins bound to MNK1 and MNK2, we discovered transcription regulation of p53 as one of the pathways significantly represented. Therefore it may be relevant in future studies to focus on the role that MNKs play in the regulation of the p53 pathway and determine the effects of the p53 mutational status on responses to MNK inhibitors.

Our data also demonstrate that the combination of Tomivosertib with Venetoclax enhances the anti-leukemic responses in mutant p53-expressing AML cell lines. The combination of these two drugs resulted in synergistic suppression of cellular viability and CFU-L growth in U937 and KG-1 cells. It has recently been proposed that AML cells can develop resistance to BH3 mimetics, such as Venetoclax, through increased levels of the anti-apoptotic protein MCL-1 via regulation of MCL-1 expression and stability [45, 46, 63, 64]. Moreover, studies involving the use of Venetoclax and MEK inhibitors to simultaneously block BCL-2 and MAPK pathways have resulted in synergistic induction of apoptosis and suppression of cell proliferation in AML models [65]. The precise mechanism for the synergistic effect of Tomivosertib and Venetoclax remains to be elucidated in the future.

It is well-established that MNK1/2 drives mRNA translation through phosphorylation of its thoroughly-characterized substrate eIF4E [15–17]. Phosphorylation of eIF4E by MNK1/2 has been shown to promote cap-dependent translation and the nuclear export of mRNAs with oncogenic potential [26–31]. However, only a few other MNK1/2 substrates are known beyond eIF4E. We identified several potential novel interactors to both MNK1 and MNK2 in our LC/MS/MS analysis which require future validation studies to determine if they are bona fide substrates or regulatory partners. Notably, our LC-MS/MS studies identified mTOR and RAPTOR as interacting partners of MNK2. We demonstrated an interaction between MNK2, mTOR and RAPTOR through co-immunoprecipitation. A previous study has shown that MNK1/2 binds to mTORC1 and helps regulate mTORC1 substrate binding [66]. We only observed mTOR and RAPTOR binding to MNK2 in our LC/MS/MS analysis which supports previous published data that MNK2 binds with much more affinity than MNK1 [66]. This also supports previously published data that MNK2, not MNK1, is responsible for the activation of the feedback loop induced by the mTORC1 inhibitor rapamycin and that MNK2 plays a role in rapamycin insensitive mTORC1 complexes [67, 68]. We did not see an effect on binding of RAPTOR to MNK2 upon MNK kinase inhibition with Tomivosertib similarly to what was observed with another MNK inhibitor CGP 57380 [66]. Overall, our data further supports previously published data that MNK proteins may play a role in regulating mTORC1.

Viewed altogether, these studies indicate that MNK1/2 inhibition would most likely be a successful strategy in only a subset of AML patients. In future studies it will be crucial to ascertain what pathways are responsible for sensitivity to MNK inhibitors. These studies will help to identify potential regulatory programs through which MNK1/2 modulates cell signaling pathways critical for leukemic cell survival and may lead to the development of novel therapeutic interventions for AML.

## MATERIALS AND METHODS

### Chemical reagents

Tomivosertib (eFT-508) and Venetoclax (ABT-199) were purchased from TargetMol. All compounds were dissolved in dimethyl sulfoxide (DMSO) and used at the indicated doses.

### Cell lines

All cells were cultured as previously described [69–73, 77] and were authenticated through short tandem repeat (STR) profiling at least once per year.

### Clonogenic leukemic progenitor assays in methylcellulose

Clonogenic assays in methylcellulose were conducted as in previous studies [69–73, 77]. For AML patient samples, informed consent was obtained prior to collection of peripheral blood or bone marrow cells as approved by the Institutional Review Board of Northwestern University.

### Cell viability assays

Cell viability assays were performed and analyzed as previously reported using WST-1 reagent (Roche) [73–75, 77].

### Cell lysis and immunoblotting

Cells were treated with either DMSO (vehicle-control) or Tomivosertib at the indicated doses and time points and processed for immunoblotting using an enhanced chemiluminescence method as described previously [69–75, 77]. Antibodies against phosphorylated-eIF4E at S209 (p-eIF4E (Ser209) (Cat. No. 9741) and mTOR (Cat. No. 4517) were purchased from Cell Signaling Technology. Antibodies against eIF4E (Cat. No. sc-9976) and HSP-90 (Cat. No. sc-7947) were purchased from Santa Cruz Biotechnology. Antibody against MNK2 (Cat. No. 17354-1-AP) was obtained from Proteintech. Antibody against RAPTOR (05-1470) was purchased from EMD Millipore. Anti-HA-tag HRP-conjugated (Cat. No. 14031) antibody was purchased from Cell Signaling Technology. Anti-FLAG-M2 HRP-conjugated (Cat. No. A8592) antibody was obtained from Sigma Aldrich.

### Plasmids and transfections

MNK1 and MNK2a cDNAs were subcloned from pMX-puro (kindly provided by Dr. Rikiro Fukunaga, Osaka University) into pcDNA3.1-FLAG plasmid. pRK5-HA-RAPTOR [76] was purchased from Addgene. 293T cells were transfected using lipofectamine 2000, as stated by the manufacturer’s protocol.

### Co-immunoprecipitation assays

Cells were treated with either DMSO (vehicle-control) or Tomivosertib at the indicated doses and time points. Samples were processed and immunoprecipitation was performed using anti-FLAG-M2 agarose conjugated beads (Sigma-Aldrich) or anti-HA Sepharose conjugated beads (Cell Signaling) as previously described [77].

### Proteomics immunoprecipitation analysis using LC-MS/MS

Samples were prepared and processed by LC-MS/MS analysis as previously reported [77]. Immunoprecipitation was performed with anti-FLAG-M2 agarose conjugated beads.

### Gene annotation and protein function enrichment analysis

This was performed as previously described using the Metascape database [77, 78].

### Statistical analyses

All experiments were performed in triplicate and repeated at least twice; variations about mean were presented as standard error. One-way ANOVA was used to compare more than two groups followed by Tukey’s multiple comparison test. Student’s *t*-test was used to assess differences between two groups. Differences were considered statistically significant when *p* values were less than 0.05. All statistical analyses were performed using Prism GraphPad 6.0.
